# Hyaluronan Synthase 1: A Mysterious Enzyme with Unexpected Functions

**DOI:** 10.3389/fimmu.2015.00043

**Published:** 2015-02-05

**Authors:** Hanna Siiskonen, Sanna Oikari, Sanna Pasonen-Seppänen, Kirsi Rilla

**Affiliations:** ^1^Department of Dermatology, Kuopio University Hospital, University of Eastern Finland, Kuopio, Finland; ^2^Institute of Biomedicine, University of Eastern Finland, Kuopio, Finland

**Keywords:** hyaluronan, hyaluronan synthase, CD44, inflammation, cytokines, cancer

## Abstract

Hyaluronan synthase 1 (HAS1) is one of three isoenzymes responsible for cellular hyaluronan synthesis. Interest in HAS1 has been limited because its role in hyaluronan production seems to be insignificant compared to the two other isoenzymes, HAS2 and HAS3, which have higher enzymatic activity. Furthermore, in most cell types studied so far, the expression of its gene is low and the enzyme requires high concentrations of sugar precursors for hyaluronan synthesis, even when overexpressed in cell cultures. Both expression and activity of HAS1 are induced by pro-inflammatory factors like interleukins and cytokines, suggesting its involvement in inflammatory conditions. Has1 is upregulated in states associated with inflammation, like atherosclerosis, osteoarthritis, and infectious lung disease. In addition, both full length and splice variants of HAS1 are expressed in malignancies like bladder and prostate cancers, multiple myeloma, and malignant mesothelioma. Interestingly, immunostainings of tissue sections have demonstrated the role of HAS1 as a poor predictor in breast cancer, and is correlated with high relapse rate and short overall survival. Utilization of fluorescently tagged proteins has revealed the intracellular distribution pattern of HAS1, distinct from other isoenzymes. In all cell types studied so far, a high proportion of HAS1 is accumulated intracellularly, with a faint signal detected on the plasma membrane and its protrusions. Furthermore, the pericellular hyaluronan coat produced by HAS1 is usually thin without induction by inflammatory agents or glycemic stress and depends on CD44–HA interactions. These specific interactions regulate the organization of hyaluronan into a leukocyte recruiting matrix during inflammatory responses. Despite the apparently minor enzymatic activity of HAS1 under normal conditions, it may be an important factor under conditions associated with glycemic stress like metabolic syndrome, inflammation, and cancer.

## Introduction

Hyaluronan is the most abundant matrix polysaccharide, which maintains tissue homeostasis, gives compressive strength for tissues, acts as an ideal lubricant in body fluids and accelerates growth and healing. In addition, excess hyaluronan promotes cancer progression and mediates inflammation. Therefore, membrane-bound hyaluronan synthases (HAS1–3), special enzymes responsible for hyaluronan production, have a key role in regulation of these conditions. Despite highly homologous amino acid sequences, HAS’s differ in subcellular localization, enzymatic activity, and regulation (1).

Despite almost 20 years of active research to sequence hyaluronan synthase genes, it is not known why vertebrates have three different isoforms of these enzymes, which are coded by separate genes on different chromosomes, to synthesize a single sugar polymer. Most research has focused on HAS2 and HAS3, while HAS1 has received the least attention and remains the most enigmatic, with only a few published reports of its biological effects on cellular behavior or association with disease states.

Knocking out the activity of hyaluronan synthase genes has provided a better understanding about normal HAS function. Knockout of *Has2* results in embryonic lethality with severe cardiac and vascular malformations (2), while the knockout of *Has1* or *Has3* does not have any apparent phenotype under non-stressed conditions (3, 4). However, double knockout of *Has1* and *Has3* leads to enhanced inflammation and accelerated wound closure of mouse skin (5), suggesting that they are necessary for the regulation of acute inflammation following injury.

A number of recent studies have highlighted the role of HAS1 in health and disease. Interestingly, *Has1* was the most upregulated gene in aneuploid mouse embryonic fibroblasts (MEFs) with malignant properties (6) and splice variants of *HAS1* are suggested to contribute to genetic instability (7), suggesting that it is susceptible to genetic alterations during oncogenic transformation. Surprisingly, HAS1 immunostainings of breast carcinoma cells correlated with hyaluronan staining, estrogen receptor negativity, HER2 positivity, high relapse rate, and short overall survival. In stromal cells of tumors from the same patients, the staining level of HAS1 was related to obesity and large tumor size (8). Human mesenchymal stem cells from different donors express *HAS1* in variable but significant levels (9), suggesting its contribution to formation of a hyaluronan niche that maintains stemness of the cells. *HAS1* is upregulated during human keratinocyte differentiation (10) and its expression correlates with levels of HA synthesis, indicating that HAS1 is an important regulator of skin homeostasis. Furthermore, as compared to other isoforms, differences in HAS1 substrate requirements (11–13), subcellular localization, and the structure of the hyaluronan coat (7, 13, 14) have been reported, suggesting an independent role of HAS1 in the regulation of cell and tissue homeostasis. However, a comprehensive review of HAS1 has not been published. Therefore, the purpose of this review is to summarize and discuss the current knowledge of this mysterious enzyme. In this review, the abbreviations *Has1* and Has1 are used for non-human gene and protein, and *HAS1* and HAS1 for human gene and protein, respectively.

## Genetics and Function of Has1 Genes and Proteins

Hyaluronan is synthesized by HAS enzymes found in vertebrates, some bacteria, and a virus (15). The first *Has* was cloned in Group A *Streptococcus pyogenes* and it was predicted to be an integral membrane protein (16). The first human *HAS* gene was isolated by two research groups almost simultaneously. Shyjan and co-workers used functional expression cloning in Chinese hamster ovary (CHO)-cells (17) and Itano and Kimata screened cDNA libraries of human fetal brain (18).

Mammalian cells have three distinct synthase genes, *Has1-3* (the human genes are abbreviated here as *HAS1-3*). They are well-conserved with highly homologous amino acid sequences, but located on separate chromosomes. In humans, *HAS1* resides in chromosome 19 at q13.3–13.4, *HAS2* is located in chromosome 8 at q24.12 and *HAS3* is in chromosome 16 at q22.1 (19). *HAS1* gene has five exons, whereas *HAS2* and *HAS3* both have four (20). Several alternative splice variants of *HAS1* have been reported in Waldenström’s macroglobulinemia (21), multiple myeloma (22), and bladder cancer (23). *In silico*, the *HAS1* gene has 46 possible transcription-factor binding sites 500 bp upstream of the transcription start site (20).

*Has1* is not essential for embryogenesis. *Has2* knockout mice die at embryonic day 9.5 due to cardiovascular defects (2), but mice deficient in *Has1* (3) or *Has3* (4) are viable and fertile. Furthermore, double knockout *Has1* and *Has3* mice have been developed and are phenotypically normal (5).

The three hyaluronan synthase proteins in humans are designated as HAS1, HAS2, and HAS3. Mammalian hyaluronan synthases are integral membrane proteins with 4–6 transmembrane domains in addition to 1–2 membrane-associated domains (15, 24). The synthase enzymes need Mg^2+^ or Mn^2+^ to produce hyaluronan, in addition to the uridine diphosphate (UDP) sugar precursors, UDP–glucuronic acid (UDP–GlcUA), and UDP–*N*-acetylglucosamine (UDP–GlcNAc) (15, 25) The synthesis takes place at the inner surface of the plasma membrane utilizing cytoplasmic precursors (26). Human and mouse enzymes add the precursor sugars to the reducing end of the growing polymer (27–29), while amphibian *Xenopus laevis* Has utilizes the non-reducing end (30), like the *Pasteurella multocida* hyaluronan synthase (31).

It has been suggested that the HAS enzymes do not require any primers for the synthesis of hyaluronan (32). The adenosine triphosphate-binding cassette (ABC) transporters have been proposed to be important for hyaluronan translocation on the plasma membrane of fibroblasts (33), requiring a concurrent efflux of K^+^ ions (34). However, ABC transporters do not seem to contribute to the translocation of hyaluronan in breast cancer cells (35). The Has protein has been shown to produce hyaluronan in a combined process of synthesis and membrane translocation, as demonstrated by Has reconstituted into proteoliposomes in *Streptococcus equisimilis* (Se) (36). In addition, there is an intraprotein pore in Has and the synthase itself is able to translocate hyaluronan in liposomes containing purified *Se*-Has (37).

## Regulation of HAS1 Expression and Activity

The three *HAS* genes are often regulated in parallel (38, 39) and the synthesis of hyaluronan reflects changes at the mRNA level (40–44). *HAS1* expression is transcriptionally regulated by transforming growth factor-β (TGF-β) in synoviocytes (45, 46) and by the pro-inflammatory cytokine interleukin-1β (IL-1β) in fibroblasts (44, 47, 48), while these factors may have similar or opposite effects on other *HAS*s, depending on cell type. The nuclear factor kappa B (NF-κB) (49) and tyrosine kinases (50) have been shown to be important for IL-1β-induced *HAS1* activation, while induction of *HAS1* by TGF-β seems to act through the p38 MAPK pathway (51). There is evidence that some of the effects are mediated by transcription-factors sp1 (52) and sp3 (53). Table 1 summarizes the growth factors and cytokines that regulate *Has1/HAS1* expression. In addition to these factors, ultraviolet B radiation induces a fast up-regulation of *Has1* expression in rat epidermal keratinocytes (54). Additionally, *Has1* expression levels are raised in renal (55) and pulmonary (56) ischemia and hyperglycemia (57). The synthesis of hyaluronan by HAS1 is also regulated by the substrate concentrations of the precursor sugars (discussed in detail later in this review).

**Table 1:** **Transcriptional regulation of *Has1/HAS1* by different growth factors and cytokines (↑ increased, ↓ decreased)**.

Agent	Cell/tissue	HAS1	Reference
EGF	Human fibroblast	↑	(44)
EGF	Human oral mucosal cell	↑	(44)
FGF2	Human dental pulp	↑	(58)
FGF2	Human periodontal ligament	↑	(59)
FGF	Human fibroblast	↑	(60)
Forskolin	Human orbital fibroblast	↑	(48)
IGF	Human fibroblast	↑	(60)
IL-1β	Human fibroblast	↑	(44)
IL-1β	Human fibroblast	↑	(61)
IL-1β	Murine uterine fibroblast	↑	(47)
IL-1β	Human orbital fibroblast	↑	(48)
IL-1β	Human dermal fibroblast	↑	(53)
PDGF	Human fibroblast	↑	(62)
Progesterone	Murine uterine fibroblast	↓	(47)
Prostaglandin D2	Human orbital fibroblast	↑	(63)
Prostaglandin E2	Human synoviocyte	↑	(64)
TGF-β	Human fibroblast	↑	(65)
TGF-β	Human keratinocyte	↑	(65)
TGF-β	Human synoviocyte	↑	(46)
TGF-β	Human synoviocyte	↑	(45)
TGF-β	Human dermal fibroblast	↑	(53)
Estradiol	Human vascular smooth muscle cell	↓	(66)
4-MU	Human aortic smooth muscle cell	↓	(67)
TGF-β1	Human synoviocyte	↓	(68)
TGF-β	Human mesothelial cell	↑	(40)

There is evidence that the activities of HAS2 and HAS3 are regulated by posttranslational modifications like phosphorylation (38, 69), ubiquitination (70), or O-GlcNAcylation (71). Whether these modifications are involved in the regulation of HAS1 activity is not completely known. Phosphorylation seems not to regulate HAS1 activation (72), but HAS1 can exist in multimers of full length-HAS1 or its variants, formed by intermolecular disulfide bonds (73).

The reported length of hyaluronan polymers produced by each of the mammalian Has differs, but the obtained results vary depending on the experimental set-up (74–77). For example, in membrane preparations from CHO-cells transfected with recombinant Has isoforms, Has2 produced the largest hyaluronan (over 3.9 × 10^6^ Da), Has3 produced intermediate length hyaluronan (0.12–1 × 10^6^ Da), and HAS1 produced the smallest polymer (0.12 × 10^6^ Da). However, all isoforms produced high molecular weight hyaluronan (3.9 × 10^6^ Da) in live cells (76). The size of the growing hyaluronan chain is increased or decreased by mutation of certain cysteine or serine amino acids in the Has1 protein in *X. laevis*, suggesting that the size of the hyaluronan chain is affected by the ability of the synthase to bind it (74).

## Subcellular Localization and Traffic of HAS1 and Its Impact on Formation of HA-Coat

Our understanding of the localization and traffic of Has proteins has been deepened after recruitment of fluorescent HAS fusion proteins together with live cell imaging (78–80). All studies reported so far suggest that like other Has/HAS isoforms, Has1 follows the normal intracellular route from rER to Golgi (78), and its traffic is regulated similarly to other HAS isoforms (13), as shown by manipulation of its traffic in live cells by factors like 4-MU and brefeldin A (BFA).

**Figure 1:**
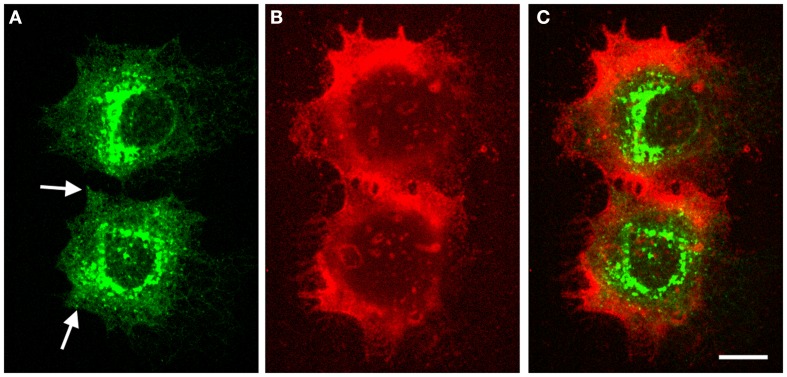
**Intracellular localization of GFP–HAS1 and structure of pericellular hyaluronan coat induced by GFP–HAS1 overexpression**. Confocal optical sections of live MCF-7 breast cancer cells transfected with EGFP–*HAS1* (green) and stained with fHABC to visualize the hyaluronan coat (red). Localization of EGFP–HAS1 is shown in **(A)**, fHABC in **(B)**, and merged images in **(C)**. Arrows in **(A)** point patches of signal near the plasma membrane. Scale bar 10 μm. Original data published in Ref. (13).

A typical subcellular localization pattern of GFP-HAS1 is presented in Figure 1. The GFP–HAS1 signal is mainly cytoplasmic, rather than on the plasma membrane, being distributed either diffusely or in cytoplasmic patches, and partially co-localizing with the Golgi apparatus (13, 14, 73). Only a small proportion of the total cellular pool of HAS1 is located on the plasma membrane, even when activated with glucosamine (12), or inflammatory cytokines like TNF-α or IL-1β (13). Occasionally, HAS1 signal is seen on or near the plasma membrane, usually as patches or concentrated spots (arrows in Figure 1), or on the plasma membrane protrusions (13, 14). The low plasma membrane signal of HAS1 is in parallel with the low activity level of HAS1, because latent HAS enzymes are thought to stay in the ER–Golgi compartment.

**Figure 2:**
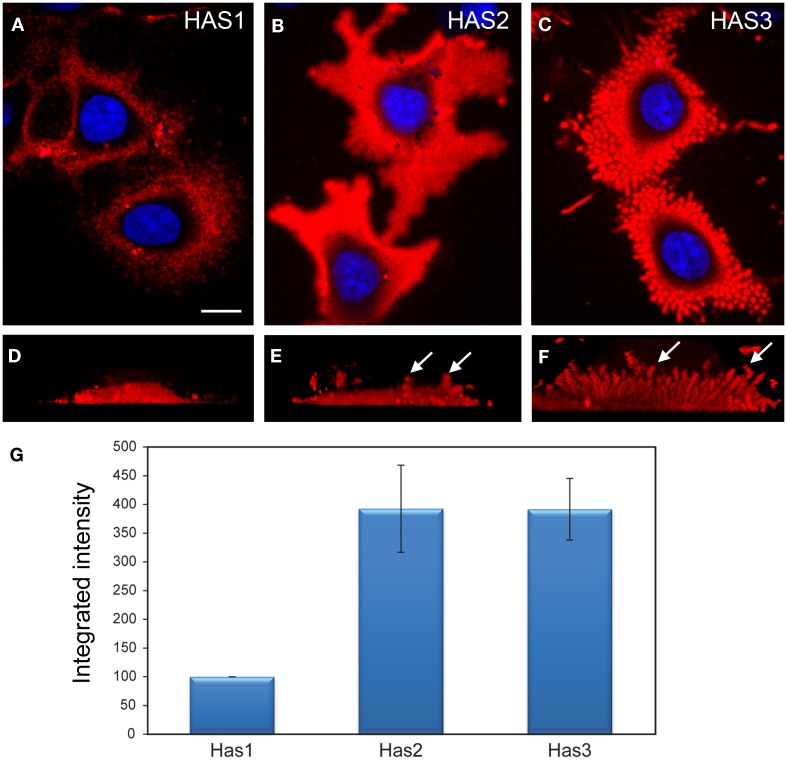
**Comparison of the structure and intensity of the pericellular hyaluronan coat in MCF-7 cells overexpressing the three HAS isoenzymes**. Structure of the hyaluronan coat of live MCF-7 cells transfected with fusion proteins Dendra2–*HAS1* **(A,D)**, Dendra2–*HAS2* **(B,E)**, and Dendra2–*HAS3* **(C,F)** and labeled with fHABC (red). Single confocal sections obtained from the middle level of nucleus (blue) are shown in **(A–C)**. Vertical views created from compressed image stacks of horizontal optical sections are shown in **(D–F)** to show the dorsal protrusions (arrows). The integrated intensity (mean intensity × area) of hyaluronan coat probed with fHABC in the three HAS transfectants was measured in thresholded area of optical sections through the center of nucleus **(G)**. Mean of three independent experiments is represented (total number of measured cells in each group = 92). Magnification bar in **(F)**, 10 μm. Original data published in Ref. (12, 81).

In addition to the full-length form, HAS1 has multiple transcript variants resulting from alternative splicing. Transfected HAS1V–GFP constructs localize with cytoskeletal structures like microtubules (7, 73). The reticular localization of the standard form of HAS1 (Figures 1 and 2) suggests that all forms of HAS1 studied so far are associated with the cytoskeletal network or endoplasmic reticulum, which is a distribution that is not typical for HAS2 or HAS3, and indicates different regulation and binding partners.

**Figure 3:**
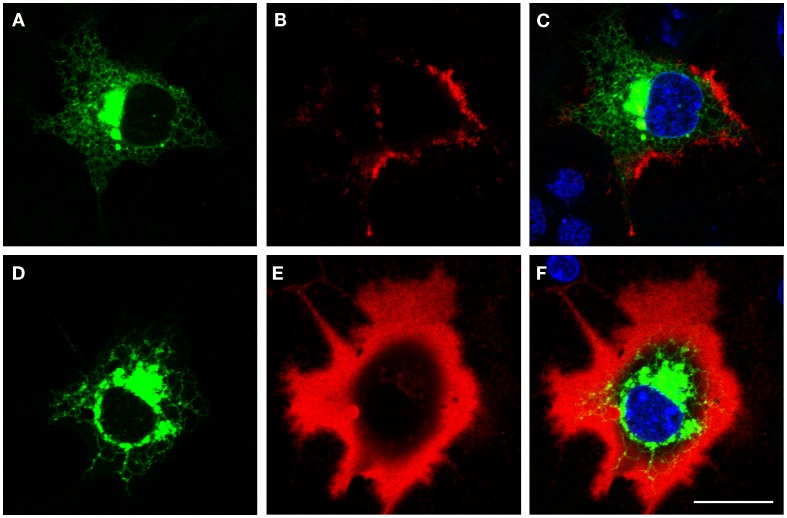
**Glucosamine induces the growth of hyaluronan coat produced by HAS1**. Confocal optical sections of pericellular hyaluronan coats on COS-1 cells expressing Dendra2–HAS1 without glucosamine **(A–C)** and after 6 h incubation with 1 mM glucosamine **(D–F)**. Green, Dendra2–HAS1; red, hyaluronan coat; blue, nuclei. Magnification bars 20 μm. Original data published in Ref. (12, 13).

The size of the pericellular hyaluronan coat correlates with activity of hyaluronan synthesis. Interestingly, even high overexpression of HAS1 in cell types with little or no endogenous hyaluronan production is not enough to produce a clearly visible hyaluronan coat (12, 13, 76). Furthermore, like previously published (12–14), the coat produced by HAS1 has a clearly different, more “cloudy” structure (Figures 1 and 2), as compared to the tight and concentrated coat around plasma membrane protrusions produced by HAS2, and especially HAS3 (Figure 2). However, the size of the coat produced by HAS1 can be induced upon induction by inflammatory agents or glucosamine (12, 13). The effect of glucosamine is presented in Figure 3. Additionally, the hyaluronan coat synthesized by HAS1 is largely dependent on hyaluronan interactions with CD44 (13).

**Figure 4:**
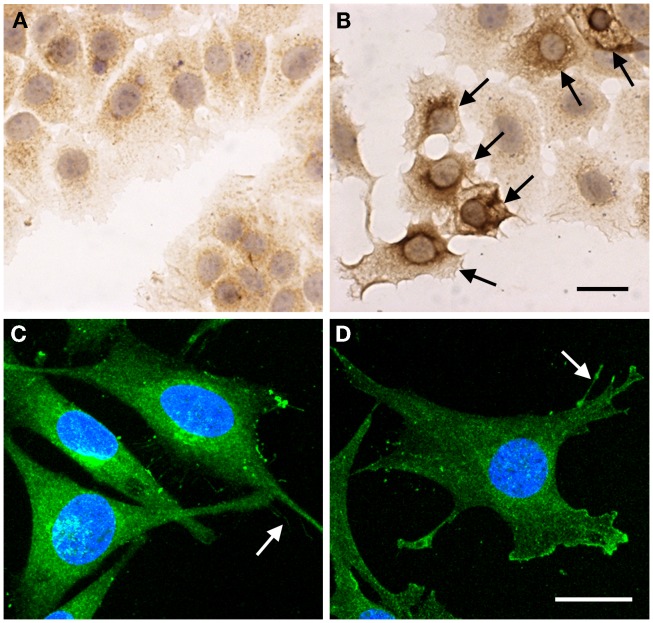
**Subcellular localization of endogenous HAS1 detected by immunostainings**. MCF-7 cells transiently transfected with empty vector **(A)** and HAS1 expressing plasmid **(B)**, followed by immunostaining with polyclonal HAS1 antibodies (brown color). Arrows in **(B)** show the HAS1 overexpressing cells. A 3D confocal projection of human chondrosarcoma cell (HCS) **(C)** and transformed mouse embryonic fibroblast (MEF) **(D)** stained with HAS1 immunofluorescence (green). Arrows in **(C,D)** point plasma membrane protrusions. Blue, nuclei. Magnification bars in **(B,D)** = 20 μm. Original data published in Ref. (6, 9, 14).

Detailed studies on tissue distribution and subcellular localization of endogenous HAS’s have been challenging due to the lack of reliable antibodies and apparently low expression level of HAS’s in many cell types. Subcellular localization of endogenous HAS1 detected with affinity purified polyclonal antibodies shows a similar pattern to exogenously expressed HAS1 fusion proteins (14). HAS1 immunostainings have shown notable levels of HAS1 in mesothelial cells, fibroblasts (14), and human chondrosarcoma cells (9). Furthermore, MEFs have prominent Has1 staining (6). Examples of HAS1 immunostainings in cultured cells are summarized in Figure 4. These results are in line with the notable mRNA levels of *Has1/HAS1* observed in these cell types (6, 12, 13).

**Figure 5:**
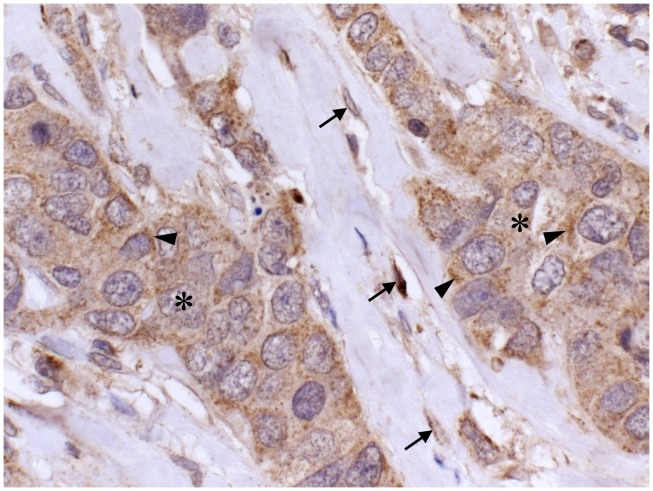
**Localization of HAS1 in breast cancer tissue**. A paraffin section of breast carcinoma immunostained with HAS1 polyclonal antibody (brown). Nuclei are labeled blue. A mainly cytoplasmic localization of HAS1 is detected in carcinoma cells (asterisks) and in stromal fibroblasts (arrows). Special accumulation of staining is seen intracellularly (arrowheads). Magnification bar 50 μm. Original data published in Ref. (8).

Staining patterns of HAS1 in tissue sections is in line with cell culture studies. Immunostainings of Has1 in developing tissues (14) and HAS1 in tumor tissues (8, 82–85), endometrium (86), and oral mucosa (87) have been published recently. In tumor tissues, HAS1 is typically expressed in tumor cells (8, 83–85), as well as in stromal fibroblasts (Figure 5). The localization of HAS1 is mainly intracellular, corresponding to the staining observed in cell cultures. Typical staining patterns vary from diffuse to granular with deposits next to the nucleus, which suggests HAS1 accumulation in the Golgi area (arrowheads in Figure 5), similar to that seen in cell cultures.

## HAS1 Requires High Cellular Content of UDP-Sugars for Activation

An important factor affecting activity of all HAS enzymes is the cytoplasmic availability of substrates, namely, UDP–GlcUA and UDP–GlcNAc. Many studies have shown that treatments influencing either UDP–GlcUA or UDP–GlcNAc levels regulate hyaluronan production [reviewed by Vigetti et al. (88)]. This role of substrates is particularly interesting in regulation of HAS1 as its activity of hyaluronan production in many cell models is low or absent unless stimulated.

In order to study the effect of UDP–GlcUA on hyaluronan production, 4-methylumbelliferone (4-MU) and overexpression of enzymes involved in either UDP-Glucose (UDP-glucose pyrophosphorylase) or UDP–GlcUA (UDP–glucose 6-dehydrogenase) production have mainly been used (39, 89, 90). These reports rely mostly on mRNA data to explain the altered hyaluronan production. The effect of UDP–GlcUA fluctuations on HAS1–3 expression levels vary considerably from one cell line to another and it is often impossible to reveal the exact role of HAS1 during these changes. A recent investigation demonstrated that availability of UDP–GlcUA can have a direct effect on HAS1 activity, as treatment of MCF-7 cells overexpressing HAS1 with an inducing agent and 4-MU significantly decrease hyaluronan coat compared to cells treated with the inducing agent only (13). It has been reported that Has1 has a lower affinity for UDP–GlcUA than other Has’s, and the *K_m_* of Has1 is about double that of Has2–3. Interestingly, availability of the other substrate, UDP–GlcNAc, did not considerably influence the *K_m_* of Has1 toward UDP–GlcUA, whereas levels of UDP–GlcUA did have a significant effect of the *K_m_* toward UDP–GlcNAc (11).

The affinity of Has1 for UDP–GlcNAc is lower than the affinity of Has2–3 as with UDP–GlcUA. The *K_m_* toward UDP–GlcNAc of Has1 is about two to three times higher that of the other Has’s. Interestingly, all Has enzymes exhibit lower affinity toward UDP–GlcNAc than for UDP–GlcUA (11). Treatments with compounds like mannose and glucosamine that regulate UDP–GlcNAc content also affect cellular hyaluronan secretion levels (12, 91). Similar to the level of UDP–GlcUA, the availability of UDP–GlcNAc influences both mRNA levels and activity of all HAS’s. The differences in substrate affinities are well demonstrated in intact cells using HAS1 overexpressing cell lines. Both COS-1 and MCF-7 cell lines have negligible endogenous hyaluronan production, and even overexpression of HAS1 enzymes does not cause prominent changes in it. Upon treatment with glucose or glucosamine, compounds that increase the amounts of hyaluronan substrates, the HAS1 enzyme is able to produce significant amounts of hyaluronan (12, 13). Furthermore, this effect of substrate availability on HAS1 activity is dose dependent (12).

The above mentioned findings on the regulation of HAS1 activity point out that although HAS1 has a minor role in total cellular hyaluronan production, it may have significant effects when induced by increased substrate availability. Since the affinity of HAS1 for its substrates is lower compared to the two other HAS’s, the fluctuations in UDP–GlcNAc and UDP–GlcUA levels can have a more significant effect on HAS1 than on HAS2–3.

## HAS1 as a Mediator in Inflammation

Many recent results suggest HAS1 may play a pivotal role during cell stress, such as inflammation. Earlier in this frontiers review series, Petrey and de la Motte comprehensively discussed the role of hyaluronan in inflammation (92). Whether hyaluronan acts as a pro- or anti-inflammatory molecule is highly dependent on its molecular size. Generally, low-molecular weight hyaluronan fragments mediate pro-inflammatory responses (93) such as recruitment of macrophages and other leukocytes to the injured or inflamed tissue (94, 95) and stimulate transcription of genes related to inflammation including several cytokines and matrix metalloproteinases (96). Growth factors and pro-inflammatory cytokines (Table 1) released during inflammation, like TGF-β, IL-1β, and TNF-α, which stimulate inflammatory cells also induce expression of *HAS1* (44, 45, 64) and *Has1* (97). Expression of *HAS1* is also upregulated in response to prostaglandins (98, 99). Therefore, *Has1/HAS1* up-regulation has been noted in many diseases associated with inflammation such as murine atherosclerosis (100), human osteoarthritis (101), murine infectious lung disease (102), and human rheumatoid arthritis (45). *HAS1* expression is also increased, among several other genes, in osteophytic chondrocytes (103). Interestingly, the expression of both *HAS1* and *HAS2* was reduced in the synovium of patients with osteoarthritis or rheumatoid arthritis compared to healthy controls (104). Moreover, elevated HAS1 expression is observed in oral lichen planus, which is a chronic inflammatory disease of the oral mucosa (87). It is worth noting that in oral lichen planus the increased HAS1 expression is detected in the basal layers of the epithelium, which is the most affected, inflamed area in lichen planus.

It is not known whether the product of HAS1 enzyme of certain polymer length, HAS1 enzyme itself or hyaluronan with HAS1 and hyaluronan binding proteins like CD44 mediate the pro-inflammatory responses. One explanation for HAS1 involvement in inflammation might be that HAS1 is associated with production of a special type of pericellular hyaluronan coat, which is pro-inflammatory. Recently, Siiskonen and co-workers showed that inflammatory agents and glycemic stress induce HAS1 to produce an expanded pericellular hyaluronan coat (13). Compared to Has3-induced hyaluronan coat, which is rather tight and formed around microvillus protrusions (105), HAS1 produces a looser, but extensive pericellular hyaluronan coat, which is dependent on CD44. In several cell types, these types of hyaluronan coats have been shown to associate with monocyte binding (106, 107). It has even shown that hyaluronan produced by Has1 binds mononuclear cells more effectively than hyaluronan produced by the two other Has enzymes (77). This could provide an explanation for the central role of HAS1 in inflammation.

In rheumatoid arthritis, the rate of hyaluronan synthesis is altered. Hyaluronan accumulates in joints affected by rheumatoid arthritis, which causes periarticular swelling and morning stiffness (108). In synoviocytes isolated from RA patients, *HAS2* and *HAS3* are constitutively activated, but *HAS1* is the gene that responds readily to pro-inflammatory cytokines like IL-1β (49) and TGF-β (45). However, IL-1β is not able to stimulate *Has1* expression in healthy synoviocytes like in type-B synoviocytes isolated from rheumatoid arthritis patients (49, 109). This IL-1β-induced *HAS1* up-regulation is dependent on the activation of the transcription-factor NF-κB (49), like many other pro-inflammatory molecules. In type-B synoviocytes, IL-1β stimulation induces the translocation of NF-κB into the nucleus, which results in up-regulation of *HAS1* mRNA expression (49). Similarly, in fibroblast-like synoviocytes, viral infection causes NF-κB activation and increased HA release due to *HAS1* up-regulation. This *HAS1* up-regulation is reversed with mitogen-activated protein kinase p38 and JNK inhibitors indicating that viral RNA activates *HAS1* through these signaling pathways (110). Moreover, *HAS1* activation is blocked with commonly used anti-inflammatory drugs, hydrocortisone, and dexamethasone, in TGF-β stimulated synoviocytes (51). In these cells, glucocorticoids block p38 activation, which results in suppressed *HAS1* expression (51). Interestingly, sodium salicylate inhibits IL-1β induced *HAS1* activation and HA release in type-B synoviocytes (64). This might explain some of the beneficial effects of sodium salicylate in the treatment of rheumatoid arthritis.

In addition to its role in rheumatoid inflammation, altered *HAS1* levels contribute to other inflammation-related states. In murine models of asthma, *Has1* mRNA is increased at an early stage, but later decreased (111, 112). In thyroid dysfunction associated with activation of the thyrotropin receptor, hyaluronan is accumulated through up-regulation of *HAS1* and *HAS2* (113). Taken together, HAS1 seems to be fundamentally involved in the inflammatory processes. However, many questions are still waiting for an answer.

## HAS1 as a Predictor of Cancer Progression

Hyaluronan content is known to be increased in many cancers, which may be altered due to hyaluronan synthase expression. Few studies have shown a direct association of HAS’s with cancer progression *in vivo*, but interestingly, HAS1 associates with tumor progression and prognostic factors in many cases. Increased expression of *HAS1* is associated with poor patient survival in ovarian cancer (114, 115), colon cancer (116), Waldenström’s macroglobulinemia (21), and multiple myeloma (22). In multiple myeloma and Waldenström’s macroglobulinemia, the occurrence of *HAS1* splice variants, rather than the full length *HAS1*, is related to cancer prognosis. *HAS1* expression is also increased in bladder cancer, correlating with increased hyaluronan levels (23), and predicting metastasis (117). In bladder cancer, HAS1 has been shown to modulate HA and CD44 levels, affecting tumor growth and progression (118). Accumulation of hyaluronan is associated with poor patient survival in breast cancer (119, 120). Recently, HAS1 and HA stainings were found to correlate with each other in breast carcinoma cells of these tumors, and HAS1 was associated with estrogen receptor negativity, HER2 positivity, high relapse rate, and short overall survival. In addition, expression levels of stromal HAS1 and HAS2 were related to obesity, large tumor size, lymph node positivity, and estrogen receptor negativity (8).

In serous ovarian tumors, *HAS1* has been shown to be very low or totally absent, whereas the levels of *HAS2* and *HAS3* mRNA or staining levels are not elevated compared to normal ovaries or benign tumors (83). Interestingly, the levels of HAS1 and HAS2 immunostainings are decreased in melanomas, correlating with reduced hyaluronan content and poor overall survival observed in these tumors (85, 121).

## Conclusion and Future Challenges

The hyaluronan coat produced by HAS1 differs from that of other isoenzymes, as shown by fluorescent hyaluronan binding probes. The flossy and loose coat is typical for cells with mesenchymal origin, like fibroblasts, mesothelial cells, synovial fibroblasts, and chondrocytes. Furthermore, as Table 1 summarizes, most of the cells that respond to cytokines or growth factors by upregulating *Has1/HAS1* levels, are of the same mesenchymal origin. Additionally, these cell types secrete active proteoglycans and other molecules participating in hyaluronan coat formation, like versican, IαI, and TSG6, which are important players in inflammation (92) and are associated with hyaluronan cables detected in fixed cells. However, other HAS’s are active in these cells, and cell types solely expressing *HAS1* are not available, making it challenging to study the specific contribution of HAS1. The most specific method so far is the artificial overexpression of fluorescently tagged HAS1 in cells with low levels of HAS enzymes (12–14).

Interestingly, HAS1 overexpression in many epithelial cell types has shown a low activity in normal culture conditions, without addition of glucosamine or inflammatory cytokines. This suggests that these cell types may lack factors that are crucial for HAS1 activity. Several studies suggest that HAS1 has a low capacity to retain hyaluronan chains on the plasma membrane, thus other molecules may be required to retain hyaluronan chains on the plasma membrane and assemble the hyaluronan coat. A potential molecule for these interactions is CD44, which seems to play a special role in the formation of the HAS1-induced coat (13).

The complexity of hyaluronan metabolism, existence of three isoenzymes, and the crucial role of HAS2 make it complicated to study the biological effects of HAS1 in animal models. Furthermore, since most human tissues and cells express all *HAS* isoforms, it is impossible to get comprehensive answers and make conclusions on the role of a single isoenzyme. Furthermore, many cells and tissues express low or negligible levels of *HAS1* mRNA. However, variable sensitivity of the methods used and other limitations may explain the low or absent *HAS1* levels detected in some cases.

Several trials have been done to solve the function and regulation of this puzzling enzyme. Evidently, HAS1 is an important regulator during inflammation and in states with altered sugar metabolism. However, contradictory results raise several new questions, which need to be resolved before we can elucidate the exact role of HAS1.

## Conflict of Interest Statement

The authors declare that the research was conducted in the absence of any commercial or financial relationships that could be construed as a potential conflict of interest.
